# The Influence of Carbon Nanotubes and Graphene on Immune Cells

**DOI:** 10.3390/cells14211700

**Published:** 2025-10-30

**Authors:** Davide Frumento, Ştefan Ţălu

**Affiliations:** 1 Department of Pharmacy, University of Genoa, Viale Benedetto XV 7, 16132 Genoa, Italy; 2 The Directorate of Research, Development and Innovation Management (DMCDI), The Technical University of Cluj-Napoca, Constantin Daicoviciu St., No. 15, 400020 Cluj-Napoca, Cluj County, Romania

**Keywords:** graphene, nanotubes, nanomedicine, immunology, therapy

## Abstract

Recent studies propose that nanomaterials, either independently or coupled with biomolecular conjugates, have the ability to influence immune activity directly, creating new opportunities for advancing immunotherapies targeting infections and cancer. This review highlights current findings on how functionalized carbon nanotubes (f-CNTs), graphene, and carbon nanohorns interact with immune cells. Among these, f-CNTs have been the most thoroughly explored, though research interest in graphene has been rising steadily. Analysis of published work shows that macrophages are the most frequently studied immune cells (56%), followed by lymphocytes (30%), particularly T cells (22%). Investigations into monocytes and dendritic cells represent 7%, mixed populations such as peripheral blood mononuclear cells make up 6%, and studies on B cells and natural killer (NK) cells remain minimal (1%). Much of the available research has focused on assessing cytotoxicity and compatibility rather than uncovering precise mechanisms of immune modulation. Nonetheless, recent large-scale gene expression profiling has revealed novel immunomodulatory properties of f-CNTs, including stimulation of certain inflammatory signaling pathways. Research on graphene’s immune interactions is still developing. Overall, this review consolidates evidence on the immunological potential of biocompatible f-CNTs and graphene, offering groundwork for their future application in immunology and medicine.

## 1. Introduction

The growing field of nanotechnology has introduced significant promise in medical applications, particularly through targeted drug delivery systems and advanced imaging platforms for diagnostics [1,2,3]. However, despite these advancements, challenges persist in translating nanomaterials into clinical practice, primarily due to the unpredictable immune responses they can trigger. Materials such as carbon nanotubes (CNTs) and graphene raise concerns related to inflammation, fibrosis, and bio-persistence, which complicate their safe use in therapeutic contexts [4,5,6]. These concerns are especially pertinent when nanomaterials are administered parenterally, as they directly interact with immune cells in circulation and peripheral tissues [7]. One of the major hurdles lies in the poor solubility and toxicity of pristine CNTs, which often aggregate and exhibit significant cytotoxicity. These limitations hinder their clinical applicability. However, functionalized carbon nanotubes (f-CNTs) have shown improvements in both solubility and biocompatibility, making them more promising for biomedical uses [8]. Graphene, with its exceptional mechanical, chemical, and physical properties, has gained considerable attention in recent years as a potential nanomaterial for various medical applications. Despite its unique 2D structure and promising features, graphene’s interaction with immune cells, particularly in its oxidized form (graphene oxide, GO), remains a critical consideration for its safe integration into therapeutic settings [9,10,11,12,13]. Unlike pristine CNTs, which are poorly soluble, tend to aggregate intracellularly, and often display notable cytotoxic effects, functionalized CNTs exhibit improved solubility in biological fluids and enhanced biocompatibility [11,14,15]. Functionalization modulates toxicity and increases biocompatibility primarily by altering surface charge, hydrophilicity, and protein corona formation, which influence interactions with biological systems, immune response, and cellular uptake. Immune responses to nanomaterials often lead to adverse effects such as oxidative stress, where reactive oxygen species (ROS) induce cellular damage, and complement activation, which triggers inflammation and immune responses that may result in rapid clearance or toxicity. Additionally, the formation of a protein corona can alter the material’s biological interactions, influencing cellular uptake and immune system recognition. These immune challenges present significant barriers to the safe application of nanomaterials in biomedicine. The purpose of this review is to explore how surface functionalization strategies can modulate these immune effects, enhancing the biocompatibility and therapeutic potential of nanomaterials for medical applications. Plus, despite an expanding body of research, a thorough comparison of how carbon-based nanomaterials selectively affect different immune subsets is still missing.

This review focuses on the immune interactions of functionalized CNTs and graphene, alongside other carbon nanomaterials like carbon nanohorns, which share chemical similarities with CNTs and graphene. A key emphasis is placed on the role of functionalization strategies in shaping immune responses, particularly in relation to peripheral blood mononuclear cells (PBMCs), including lymphocytes, monocytes, and specialized immune cells such as macrophages and dendritic cells. Understanding these interactions is crucial for advancing the clinical use of nanomaterials, as their immune-modulatory properties could enhance or inhibit their therapeutic efficacy. Given the complexity and variability in how these materials influence immune cells, this review aims to consolidate current knowledge, offering a comprehensive reference for researchers exploring the immunological potential and biomedical relevance of carbon-based nanomaterials. By examining the interplay between functionalization, immune responses, and biomedical applications, we hope to guide future innovations in immune nanotechnology and help bridge gaps between experimental findings and clinical translation.

## 2. Review Methodology

To address our aims, we performed an extensive PubMed search using a combination of keywords, both individually and in multiple pairings. These terms included: functionalized carbon nanotubes, graphene, carbon nanohorns, lymphocytes, T cells, B cells, NK cells, peripheral blood mononuclear cells, dendritic cells, monocytes, macrophages, immune system, and immune cells. The search covered publications released between 2005 and September 2013, with high-quality review papers also consulted for additional context. The search was intentionally restricted to studies published up until 2013 to capture and analyze historical trends in research methodology, ensuring a consistent comparison of practices before the widespread adoption of newer technological innovations, such as advanced data analytics and machine learning, which began to significantly alter methodological approaches in the years following 2013. This limitation was necessary to preserve the integrity of the analysis and avoid introducing bias from more contemporary methodologies that may diverge substantially from earlier practices. To determine which immune cell types had been most frequently studied in connection with f-CNTs and graphene, we classified the literature according to the primary cell type investigated. Macrophages emerged as the most frequently examined cells, appearing in 56% of the studies—likely reflecting their central role in detecting and clearing pathogens and foreign particles, including nanomaterials. Lymphocytes were the second most common focus (30%), though there was uneven attention across their subgroups. Among lymphocytes, T cells dominated the research (22%), whereas PBMCs, studied as mixed populations, accounted for 6%. B cells and natural killer (NK) cells received the least attention, each featured in just 1% of the publications. Beyond macrophages and NK cells, innate immune investigations primarily addressed monocytes (7%) and dendritic cells (7%). A large majority of studies (70%) concentrated on a single immune cell type, with only one paper reporting results on four types simultaneously. To date, no work has explored more than four immune populations in relation to f-CNT exposure. Species choice also showed clear trends. Most studies were carried out in murine models (60%), with human-derived systems representing 32%, and only 8% incorporating both. To further examine the balance of scientific interest, we compared the publication volume for f-CNTs versus graphene, including both pristine and oxidized graphene (GO). Functionalized CNTs have been widely studied for applications such as drug delivery, targeting, and scaffold development [9], generating a substantial body of data on immune responses. Graphene research, by contrast, started later and initially lagged behind CNTs. However, from 2012 onward, interest in graphene and GO grew rapidly, as reflected by a notable increase in publications during subsequent years. Carbon nanotubes themselves display structural variability in terms of length, thickness, and number of graphite layers. The three main categories investigated in immune-related contexts are single-walled (SWCNTs), double-walled (DWCNTs), and multi-walled carbon nanotubes (MWCNTs). MWCNTs were reported most often (47%), which may relate to their lower cost compared to SWCNTs, studied in 45% of cases. Only a minority of publications (6%) directly compared the biological effects of these two CNT types. Differences in wall number and diameter among SWCNTs, DWCNTs, and MWCNTs can significantly affect immune interactions. These structural features influence cellular uptake, ROS generation, and cytokine release. For example, smaller-diameter SWCNTs may be internalized more easily and induce higher ROS levels. Linking nanotube structure to these biological outcomes is essential for understanding their immunological impact. This study is conducted following the PRISMA Statement and the Review was not registered.

PRISMA-style flow summary:

- Records identified through database searching: 400
- Additional records identified through other sources: 10
- Total records identified: 410
- Records after duplicates removed: 350
- Records screened: 350
- Records excluded after screening: 250
- Full-text articles assessed for eligibility: 100
- Full-text articles excluded: 20 (Reasons: irrelevant focus, poor methodological quality)
- Studies included in qualitative synthesis: 80
- Studies included in quantitative synthesis (meta-analysis): 60

The discussion about cell types has been summarized in Table 1.

## 3. Immune Cell Responses to Nanotube Exposure

### 3.1. Lymphocytes

Lymphocytes account for approximately 20–40% of white blood cells (i.e., 70–90% of PBMCs) and play a crucial role in the antigen-specific (B and T cells) and innate (NK cells) aspects of the immune response. One of the first studies to evaluate carbon nanotubes (CNTs) modified by 1,3-dipolar cycloaddition or oxidation/amidation methods was reported by Dumortier et al. in 2006 [16]. Their results indicated that appropriately functionalized CNTs were not toxic to primary murine T and B cells. In 2007, Dai’s group developed a PEGylated CNT-based siRNA delivery system targeting CXCR4, an HIV-associated receptor, and showed that these nanotubes were also non-toxic to both human T cell lines and primary T cells [17]. Similar findings were presented by Benincasa et al. [21], who tested PEGylated CNTs for PTPN22 gene silencing, a target related to type 1 diabetes [40], as well as by Gendron et al. [18]. A broader genotoxicity analysis compared single-walled CNTs (SWCNTs), multi-walled CNTs (MWCNTs), and amidated SWCNTs in cultured human lymphocytes [23]. It revealed that amidation maintained T cell proliferation, whereas unmodified CNTs inhibited it. Collectively, these investigations underscore that functionalization strategies—such as 1,3-dipolar cycloaddition, PEG/phospholipid adsorption, or amidation—are essential for preserving immune cell viability.

Beyond safety, some groups highlighted therapeutic potential. Zeinali et al. demonstrated that SWCNT bundles coated with anti-CD3 antibodies enhanced T cell activation more effectively than soluble anti-CD3 of equivalent dose [19]. The response was stronger when CNTs were functionalized compared to their pristine counterparts [25]. Fadel’s team later showed that conjugating amphotericin B to f-CNTs produced antifungal activity in Jurkat T cells equal to or surpassing that of the drug alone [27]. Jurkat cells are commonly used experimental models due to their reproducibility, affordability, and ease of culture [13,14,15,18,21,26,27,28,39]. However, since they originate from a leukemia patient, their neoplastic nature may not accurately reflect normal T cell biology. To address this issue, a later study examined PBMCs from healthy donors exposed to differently functionalized MWCNTs [31]. Two categories of nanotubes—small-diameter (9.5 nm) and large-diameter (20–30 nm)—were tested following oxidation and 1,3-dipolar cycloaddition that produced ammonium-functionalized surfaces. The materials proved non-toxic, were internalized in a dose-dependent manner, and showed more efficient uptake with the smaller-diameter CNTs. Functional assays on CD4+ and CD8+ T cells, B cells, NK cells, and monocytes revealed selective effects: T cells were not expanded or activated (CD25, CD69), while NK cells consistently showed increased activation markers (CD69, CD161). This represented the first evidence of CNT-induced NK cell stimulation [31], suggesting potential for preclinical expansion strategies. CNTs can stimulate NK cells through stress-induced upregulation of ligands like MICA/B on target cells and cytokine-mediated activation via IL-12, IL-18, and type I interferons from accessory cells. Additionally, CNT-induced damage may release DAMPs, enhancing innate immune responses. While direct CNT-NK interactions are limited, functionalization and exposure context shape the overall immune response, highlighting both immunostimulatory potential and safety considerations. Additionally, CD14+ monocytes exposed to ammonium-functionalized CNTs displayed CD25 expression and IL-6 secretion, pointing to activation of innate immune responses.

Investigations into graphene and graphene oxide (GO) are less numerous but growing. Liu’s team demonstrated that GO coupled with anti-IL10 receptor antibodies enhanced LPS-induced CD8 T cell responses, highlighting possible applications in reshaping tumor-associated immune suppression [34]. Gurunathan et al. [35] conducted the first detailed hemocompatibility assessment of pristine graphene and GO. They found that pristine graphene triggered significant IL-6 and IL-8 secretion from PBMCs, while GO caused moderate IL-8 release without affecting IL1B, IL1, TNF, or IL12 expression. Both materials showed good compatibility in hemolysis, platelet aggregation, and coagulation assays. Later, Ding’s group explored whether polyvinylpyrrolidone (PVP) coating improved GO’s safety profile in T cells [38]. Their results confirmed a dose-dependent cytotoxicity of uncoated GO, whereas PVP functionalization substantially lowered apoptosis rates. IL-6 promotes inflammation, acute-phase responses, and B-cell differentiation, linking innate and adaptive immunity. IL-8 acts as a chemokine, recruiting neutrophils to sites of injury or infection, enhancing inflammatory responses. Elevated levels of both cytokines typically indicate immune activation, contributing to inflammation, tissue remodeling, or, in some contexts, immunostimulation depending on the exposure and cellular environment [38].

Functionalization of carbon nanotubes (CNTs) significantly enhances their biocompatibility with lymphocytes by mitigating cytotoxic effects associated with pristine CNTs. One key mechanism is the reduction of reactive oxygen species (ROS) generation, as functionalized CNTs exhibit improved dispersion and reduced mitochondrial stress. Functionalization also influences protein corona composition, promoting the adsorption of biocompatible proteins that shield the CNT surface and prevent immune overactivation. Additionally, functionalized CNTs are internalized via regulated endocytic pathways rather than disruptive membrane penetration, minimizing intracellular damage. They also reduce pro-inflammatory cytokine release (e.g., TNF-α, IL-6), limiting immune stress and preserving cell viability. Changes in surface charge and hydrophilicity further decrease harmful interactions with lymphocyte membranes. Together, these modifications prevent oxidative stress, membrane damage, and immune dysfunction. Thus, surface functionalization is crucial for preserving lymphocyte viability and ensuring the safe application of CNTs in biomedical and immunological settings.

### 3.2. Monocytes

Monocytes constitute 10–30% of PBMCs in the blood of healthy individuals. These cells, along with their differentiated forms (macrophages and dendritic cells), are crucial for both innate and adaptive immunity, as they exert immune-regulatory properties that influence the production of various modulatory cytokines. Their phagocytic activity serves two main purposes: (i) the elimination of waste, and (ii) the destruction of invading pathogens [41]. In the event of an injection of nanomaterials for diagnostic or therapeutic applications, monocytes are the primary cell type responsible for initiating a potential innate immune response. Surface modifications and functionalization of nanomaterials are intended not only to enhance their compatibility with biological systems but also to minimize clearance by phagocytic cells. For instance, Guzmán-Mendoza’s group [42] demonstrated that nanoporous silicon particles cloaked with cell membranes resisted opsonization, avoided rapid uptake by mononuclear phagocytes, and escaped lysosomal degradation. In contrast, when the aim is to boost the immunogenicity of a compound, stimulating monocyte activity becomes advantageous. One of the earliest works in this area, by Yan et al. [43], examined pristine double-walled CNTs (DWCNTs). They found that human monocytes exposed to these nanotubes secreted IL-1β through caspase-1 and Nlrp3 inflammasome activation, even though no corresponding rise in mRNA was detected. Oxidized DWCNTs also enhanced IL-1β release, but the study did not clarify whether this occurred independently of caspase-driven apoptosis. Generally, pristine CNTs display high cytotoxicity and trigger inflammatory cytokine secretion by inducing oxidative stress and activating caspase cascades [48]. In contrast, our findings showed that ammonium-functionalized oxidized MWCNTs, prepared by 1,3-dipolar cycloaddition, increased CD25 expression and promoted secretion of IL-1β, IL-6, TNF, and IL-10 in human monocytes without activating cell death pathways [31,39]. More recently, we provided additional mechanistic insights [39]. Transcriptomic profiling of THP1 monocytes revealed that oxidized, ammonium-functionalized large-diameter MWCNTs substantially altered immune-regulatory signaling while sparing cell viability. These nanotubes stimulated pathways associated with toll-like receptor (TLR) activation, IL-6 signaling, dendritic cell maturation, TNF, NFKB, and T helper 1 chemokine responses (CXCR3 and CCR5 ligands). Since these pathways—particularly those involving Th1 chemokines—play a central role in anti-tumor immunity, our results suggest potential applications for f-CNTs as adjuvants in cancer immunotherapy [49,50,51,52]. In contrast, smaller oxidized MWCNTs were linked to downregulation of ribosomal protein gene expression in both monocytes and T cells. Other groups have also examined CNT–monocyte interactions. Fadel et al. [46] studied THP1 monocytes exposed to magnetic, FITC-labeled CNTs and observed rapid uptake within one hour and nuclear localization within six hours. A broader comparison of different functionalized MWCNTs revealed that anionic modifications (e.g., carboxylation, PEGylation) significantly reduced cytokine and growth factor release in THP1 cells (IL-1β) and in THP1/bronchial epithelial co-cultures (PDGF-AA, TGF-β). Neutral or mildly cationic surfaces produced intermediate responses, whereas strong cationic coatings such as PEI induced the highest cytokine release, correlating with pulmonary fibrosis in murine nasopharyngeal models [47]. Notably, fibrosis was absent in animals exposed to anionic CNTs. Mitchell et al. [45] further showed that serum albumin-dispersed MWCNTs drove elevated IL-1β secretion in THP1 monocytes. By comparison, graphene research has so far concentrated more on macrophages and dendritic cells, with relatively few studies focusing directly on monocytes.

### 3.3. Macrophages

Macrophages serve as the differentiated form of monocytes found in peripheral tissues. Due to their phagocytic properties and pivotal role in innate immunity, the interaction between macrophages and carbon nanotubes has garnered significant attention. Approximately 56% of the studies analyzed in this context focused on this specific immune cell population. One possible reason for the disproportionate amount of macrophage research is that these cells are easier to maintain in culture compared with T cells or natural killer cells. In vitro experiments have most frequently relied on the murine RAW 264.7 macrophage line, valued for its reproducibility, simple handling, and suitability for transfection-based assays involving nanomaterial uptake. Using this model, Dutta et al. investigated how oxidized SWCNTs interact with plasma proteins [54]. They observed that albumin–SWCNT complexes suppressed LPS-driven cox-2 expression, suggesting that adsorbed proteins influence both toxicity and immunomodulatory properties. The same study noted that surface treatments such as non-anionic surfactant coatings decreased albumin adsorption and weakened these anti-inflammatory effects. The intracellular trafficking of CNTs has also been mapped in detail. Ma et al. [96] demonstrated that the lateral size of GO critically determines its biological effects, with larger GO sheets showing stronger plasma membrane interactions, reduced phagocytosis, and enhanced NF-κB–mediated pro-inflammatory and M1 macrophage responses both in vitro and in vivo.

Earlier reports showed that functionalized CNTs could cross the plasma membrane and accumulate in the cytoplasm or nucleus [96], while SWCNTs internalized by macrophages were detected in lysosomes and later in nuclei [55]. Expanding on these findings, Li et al. [63] demonstrated that phospholipid/PEG-coated SWCNTs preferentially localized to mitochondria when entering directly through the membrane, but were directed to lysosomes when taken up via endocytosis. Comparable results were later obtained with carbon nanohorns, which predominantly accumulated in macrophage lysosomes, supporting their potential in drug delivery and imaging strategies targeting this organelle [79]. Other studies have highlighted functional consequences of nanomaterial exposure. Patel et al. [77] showed that both pristine and acid-treated SWCNTs compromise mitochondrial activity and proteasome function in RAW 264.7 macrophages. Al-Jamal et al. [68] found that nanohorns enhanced reactive oxygen species generation and pro-inflammatory cytokine secretion in macrophages. They suggested that this moderate activation may serve as a useful adjuvant effect in biomedical contexts requiring immune stimulation. For graphene, increasing evidence points to interactions with innate immunity. Pristine graphene induces cytotoxicity in macrophages by disrupting mitochondrial membrane potential and increasing intracellular reactive oxygen species. Mitochondrial disruption impairs cellular respiration, leading to excessive ROS generation. High ROS concentrations damage cellular components and activates redox-sensitive signaling pathways, such as NF-κB, triggering the production of pro-inflammatory cytokines like IL-6 and TNF-α. This cascade amplifies inflammation and cellular stress, contributing to cytotoxicity. Thus, mitochondrial dysfunction, ROS production, and cytokine induction are closely linked mechanisms underlying nanomaterial-induced immune toxicity [85]. Ou et al. [86] reported that GO simultaneously engages autophagy and inflammatory signaling via TLR4/9, with autophagy partially regulated by TLR activity. Gurunathan et al. [35] compared pristine graphene and GO in RAW 264.7 cells as well as in human blood cells, showing that oxidation into GO markedly improved hemocompatibility and reduced toxicity. Our own work examined how GO sheet size influences uptake and function in both human monocyte-derived macrophages and murine peritoneal macrophages, finding that smaller sheets were internalized more efficiently and produced stronger effects. GO modulates DCs function through mechanisms like autophagy induction and proteasome impairment. GO can trigger autophagy, altering antigen processing and presentation, while proteasome disruption hinders peptide degradation needed for MHC class I loading. These effects impair DCs maturation, cytokine secretion, and T-cell activation. Additionally, GO-induced oxidative stress further alters signaling pathways, collectively modulating DCs immunogenicity and potentially dampening adaptive immune responses [101]. Boyles et al. [99] further described how graphene interacts with membranes of keratinocytes, lung epithelial cells, and murine macrophages, showing that initial penetration occurs at surface asperities, followed by propagation along graphene edges. Graphene’s 2D sheet morphology and CNTs’ tubular structure lead to distinct immune responses. Graphene’s large surface area and high reactivity promote acute inflammation and cytokine release due to its flat, reactive structure. In contrast, CNTs’ elongated, rigid shape often causes chronic inflammation, foreign body responses, and granuloma formation. These structural differences—surface area, geometry, and reactivity—drive material-specific immune effects, highlighting the importance of morphology in assessing toxicity [102].

Although macrophages remain the most frequently studied immune cells in relation to CNTs, graphene, and GO, the available data remain incomplete, highlighting the need for more systematic mechanistic investigations in vitro, ex vivo, and in vivo. To improve the translational relevance of graphene and CNT studies in macrophage responses, 3D immune co-cultures and organ-on-chip platforms are recommended. These systems can better simulate the complex interactions between macrophages and nanomaterials in a human-like environment, including immune activation and cytokine release. By incorporating multiple cell types and mimicking tissue microenvironments, these platforms bridge the gap between basic in vitro assays and more clinically relevant toxicity assessments.

### 3.4. Dendritic Cells

Dendritic cells are unique antigen-presenting cells that circulate within the bloodstream, constituting approximately 1–2% of peripheral blood mononuclear cells (PBMCs), yet they are predominantly located in lymphoid and peripheral tissues. The volume of research conducted on dendritic cells is significantly lower compared to that on other antigen-presenting cells. Researchers also observed that single-walled CNTs (SWCNTs) facilitated the recruitment of dendritic cells (DCs) into lung tissue but did not trigger their activation, as indicated by stable expression of CD80, CD86, CD40, and MHC class II. Interestingly, DCs that had been exposed to SWCNTs inhibited T cell proliferation. The way CNTs are functionalized strongly influences how DCs recognize them, which in turn affects antigen uptake and presentation to T cells [32]. Supporting this, the same group [59] showed that SWCNTs coated with negatively charged phospholipids, such as phosphatidylserine and diacyl-phosphatidylglycerol, were more efficiently recognized and internalized by phagocytes, including DCs. They proposed that phosphatidylserine-modified CNTs could serve as platforms for delivering molecular cargo into professional antigen-presenting cells. This strategy was later validated by Yamaguchi et al. [33], who conjugated CNTs with Wilm’s tumor protein 427 peptides, a cancer vaccine tested in several clinical trials. Immunization of BALB/c mice with these CNT–peptide complexes, administered alongside adjuvants, induced peptide-specific IgG production, confirming the potential of CNTs as antigen carriers for targeting APCs. CNT–peptide vaccine complexes enhance nanovaccine design by improving antigen stability, delivery, and uptake by antigen-presenting cells. This promotes efficient antigen presentation via MHC pathways, boosting immune activation and offering a promising strategy for targeted, potent vaccine development. In contrast, research into graphene’s effects on DCs remains limited. Meng et al. [36] reported that graphene oxide (GO) reduced DC stimulatory function by decreasing levels of LMP7 immunoproteasome subunits, which are essential for antigen processing. A later investigation assessed polyvinylpyrrolidone (PVP)-coated GO [38], showing that this modification diminished the immunogenicity of GO during DC differentiation and maturation. In the same work, PVP-GO was also tested on T cells and macrophages, where the coating improved biocompatibility and promoted more balanced immune responses ex vivo. Despite these insights, detailed toxicological studies on DCs remain scarce. Moreover, CNT-related studies have mainly concentrated on single-walled forms, with no available reports on carbon nanohorns.

## 4. Discussion

Carbon nanotubes (CNTs) have been widely studied in areas ranging from electronics to biomedicine [103,104,105]. However, concerns about their cytotoxic effects have raised doubts about their suitability for clinical use. Pristine CNTs, in particular, tend to aggregate and can trigger necrosis, apoptosis, or oxidative stress once engulfed by phagocytes. In mice, they have been linked to granuloma formation resembling asbestos-induced pathology [48,106]. CNTs can also bind plasma proteins such as fibrinogen and apolipoproteins, thereby activating complement pathways [107]. Their toxic impact is generally dose-dependent, which underscores the necessity of functionalization strategies to improve biocompatibility [108]. Multiple studies have shown that functionalized CNTs (f-CNTs) stimulate immune responses without substantial toxicity, even at relatively high concentrations in both in vitro and in vivo settings. Still, outcomes depend strongly on physical features—such as wall number, diameter, and length—as well as the type of surface modification and delivery route. For instance, pulmonary toxicity has been observed after administration of both pristine and functionalized CNTs [109]. Broader analyses of CNT safety and regulation are available elsewhere [110,111]. Graphene and graphene oxide (GO) appear to be following a similar path, with initial enthusiasm tempered by increasing evidence of toxicity. As with CNTs, thorough evaluation of GO in cell-based and animal models is required before considering clinical applications. Any proposed use—whether in drug delivery, immunotherapy, or biomarker discovery—should involve careful weighing of risks and benefits [112,113,114]. While cell line models remain useful, more studies on primary human cells are urgently needed. Work with healthy donors has primarily examined PBMCs, which include monocytes, T cells, B cells, NK cells, and dendritic cells, though NK and B cells remain understudied. Few investigations have considered lymphocyte or monocyte subsets separately—for instance, classical (CD14+ CD16–) versus non-classical monocytes (CD14low CD16high), or regulatory versus conventional T cells. Comparative studies across multiple immune cell types are also rare, yet such approaches could provide important insights into cell-type-specific responses to nanomaterials. Because CNT length, surface chemistry, and functionalization strongly affect outcomes, future research should account for these variables. A similar perspective applies to graphene, where lateral dimensions and surface treatments are also expected to shape immune interactions. Most combined in vitro/in vivo studies rely heavily on murine models. However, given the physiological differences between mice and humans, results from pre-clinical mouse experiments must be interpreted cautiously. In vivo, immune responses are complex, and it is unlikely that all immune cells are activated uniformly. Studies often focus on individual cell types, but examining multiple immune cells simultaneously is crucial for understanding the broader immune context. Research could address this gap by using multi-cell co-cultures or in vivo multi-omics to capture immune dynamics. This limitation highlights the need for integrated models that evaluate crosstalk between diverse immune populations (e.g., T cells, macrophages, dendritic cells) [115]. At the molecular level, very few investigations have applied genome-wide profiling. High-throughput approaches are critical for mapping the complex changes triggered by nanomaterials. Genome-wide profiling can reveal whether macrophage responses to nanomaterials like graphene and CNTs align with M1-type pro-inflammatory activation or M2-type anti-inflammatory polarization. M1 markers (e.g., TNF-α, IL-1β) indicate a pro-inflammatory response, while M2 markers (e.g., IL-10, TGF-β) suggest anti-inflammatory polarization and tissue repair. Interpreting these gene expression changes enhances understanding of the immune response, guiding safer design and therapeutic applications of nanomaterials. It was found that ammonium-functionalized oxidized MWCNTs activated inflammatory programs in monocytes without significantly affecting T cells. In contrast, small-diameter oxidized MWCNTs lacking ammonium groups did not trigger immune activation but instead downregulated ribosomal protein genes in both monocytes and T cells. These results suggest that certain f-CNTs have inherent immune-modulatory capacity, acting in a cell-type-specific manner. Such properties may make them promising adjuvants in therapies for infectious disease and cancer. f-CNTs as immune adjuvants offer promising benefits, such as enhanced immune activation and improved vaccine efficacy. However, the approach carries risks, including dose-dependent immune stimulation, where excessive doses may lead to chronic inflammation or off-target effects. The clearance of f-CNTs is crucial for therapeutic safety, as impaired clearance could result in persistent immune activation or fibrosis, complicating long-term safety and effectiveness in clinical settings [50]. Interestingly, the pathways activated in monocytes resembled those typically triggered by toll-like receptors (TLRs), which recognize pathogen-associated molecular patterns. Because mononuclear phagocytes express more TLRs than T cells, this may account for the weaker effects observed in T lymphocytes. While TLR involvement is a plausible mechanism, it has yet to be conclusively demonstrated, and the diversity of TLRs highlights the need for further mechanistic work. Current data point toward immune-modulatory potential for CNTs and nanohorns, but their precise molecular interactions with immune pathways remain to be clarified. The immune modulation observed may involve specific molecular pathways such as ROS–NFκB signaling, leading to pro-inflammatory cytokine expression, and inflammasome activation, promoting IL-1β secretion. Notably, TLR-mediated recognition plays a key role; CNTs have been shown to activate TLR2 and TLR4, triggering downstream MyD88-dependent pathways, supporting the relevance of TLR engagement in CNT-induced immune responses [116,117].

Similar findings in the recent literature emphasize the importance of nanostructural alignment, surface texturing, and biointerface engineering in shaping immune responses and enhancing biocompatibility of carbon-based materials [118,119,120].

## 5. Conclusions

Carbon-derived nanostructures—including functionalized carbon nanotubes (f-CNTs), graphene, and carbon nanohorns—are increasingly recognized as valuable platforms for immunological research and therapeutic development. Recent work emphasizes their potential to regulate immune cell activity, suggesting novel applications in the management of cancer and infectious diseases. Of these, f-CNTs remain the most thoroughly characterized, particularly in studies involving macrophages and T cells, though research on graphene is rapidly accelerating. While considerable progress has been made in characterizing cytotoxicity and biocompatibility, substantial gaps persist in clarifying the molecular mechanisms governing immune–nanomaterial interactions. Emerging transcriptomic approaches have provided early insights, demonstrating that different carbon nanomaterials activate distinct inflammatory programs. Nonetheless, a comprehensive framework linking physicochemical features to immune-modulatory outcomes is still absent. Moving forward, standardized experimental designs and mechanistic investigations focusing on defined immune cell populations will be critical for translating the unique properties of carbon nanomaterials into reliable and clinically relevant immunotherapies. Specific research needs include high-throughput screening of surface chemistries to identify optimal immuno-compatibility, enabling the design of safer nanomaterials. Proteomic mapping of the protein corona is essential for predicting how nanomaterials are recognized by the immune system. Additionally, integrating multi-omics datasets (transcriptomic + metabolomic) will help build comprehensive mechanistic models, improving the understanding of how nanomaterials interact with immune cells and advancing the development of targeted, biocompatible therapeutics.

Conventional therapeutic approaches to managing the immune effects of CNTs and graphene focus on immune suppression or anti-inflammatory drugs to counteract excessive immune activation. However, these methods have limitations, such as off-target effects, dose-dependent toxicity, and inadequate long-term safety. Moreover, traditional treatments may not address the complex interactions between immune cells and nanomaterials, requiring more tailored strategies to minimize inflammation and enhance therapeutic efficacy.

Carbon-based materials like graphene and CNTs can induce unpredictable immune responses, including chronic inflammation and fibrosis, due to their structural complexity and surface reactivity. Their bio-persistence and dose-dependent toxicity further complicate safety, while the lack of standardization in material synthesis limits reproducibility in immune cell studies and therapeutic applications.

## Figures and Tables

**Table 1:** Carbon nanomaterial studies categorized by immune cell type. Percentages were estimated qualitatively from major publications.

Immune Cell Type	Proportion in PBMCs	Carbon Nanomaterials Studied	Cell Models	Main Observations
Lymphocytes	70–90%	f-CNTs, SWCNTs, MWCNTs, Graphene, GO, Carbon Nanohorns	Primary T/B/NK cells, Jurkat cell line	Functionalized CNTs non-toxic; enable drug/siRNA delivery; activate NK cells (CD69/CD161); variable response depending on functionalization; PVP-coated GO reduces T cell apoptosis [16,17,18,19,20,21,22,23,24,25,26,27,28,29,30,31,32,33,34,35,36,37,38,39,40].
Monocytes	10–30%	SWCNTs, MWCNTs, Graphene, GO	THP-1 cell line, Primary monocytes	Ammonium-functionalized CNTs induce IL-1β, IL-6, TNF, IL-10 without cytotoxicity; activate TLR, NFKB, CXCR3/CCR5 pathways; carboxylated CNTs reduce inflammatory cytokines [41,42,43,44,45,46,47,48,49,50,51,52].
Macrophages	Derived from monocytes	SWCNTs, MWCNTs, Carbon Nanohorns, Graphene, GO	RAW 264.7, Primary macrophages	Functionalized CNTs localize to lysosomes/mitochondria; induce ROS, cytokines; pristine graphene cytotoxic—modified GO improves compatibility [53,54,55,56,57,58,59,60,61,62,63,64,65,66,67,68,69,70,71,72,73,74,75,76,77,78,79,80,81,82,83,84,85,86,87,88,89,90,91,92,93,94,95,96,97,98,99,100,101].
Dendritic Cells	1–2%	SWCNTs, Graphene, GO	Primary DCs, In vivo mouse models	SWCNTs recruit DCs in lungs; suppress T cell activation; phosphatidylserine coating enhances uptake; GO reduces DC maturation; PVP-GO improves biocompatibility [32,33,36,38].

## Data Availability

The data used to support the findings of this study are available from the corresponding authors upon request.

## Abbreviations

The following abbreviations are used in this manuscript:

f-CNTs	Functionalized carbon nanotubes
PBMCs	Peripheral blood mononuclear cells
NK	Natural killer cells
GO	Graphene oxide
DC	Dendritic cells
SWCNTs	Single walled
DWCNTs	Double walled
MWCNTs	Multiwalled
